# Regulation of Endoplasmic Reticulum Stress by Empagliflozin in Doxorubicin‐Induced Cardiotoxicity in Rats

**DOI:** 10.1111/jcmm.71161

**Published:** 2026-04-29

**Authors:** Akshi Malik, David C. Y. Cheung, D. Allison Ledingham, Danielle Desautels, Joerg Herrmann, Pawan K. Singal, Davinder S. Jassal

**Affiliations:** ^1^ Institute of Cardiovascular Sciences, St. Boniface Hospital Albrechtsen Research Centre Winnipeg Canada; ^2^ Department of Physiology and Pathophysiology University of Manitoba Winnipeg Canada; ^3^ Section of Hematology and Oncology, Department of Internal Medicine, Max Rady College of Medicine, Rady Faculty of Health Sciences University of Manitoba Winnipeg Canada; ^4^ Paul Albrechtsen Research Institute, CancerCare Manitoba Winnipeg Canada; ^5^ Department of Cardiovascular Medicine Mayo Clinic Rochester Minnesota USA; ^6^ Section of Cardiology, Department of Internal Medicine, Max Rady College of Medicine, Rady Faculty of Health Sciences University of Manitoba Winnipeg Canada; ^7^ Department of Radiology, Max Rady College of Medicine, Rady Faculty of Health Sciences University of Manitoba Winnipeg Canada

**Keywords:** apoptosis, doxorubicin‐induced cardiomyopathy, Empagliflozin, endoplasmic‐reticulum stress

## Abstract

Although Doxorubicin (Dox) is an effective anticancer drug, it can cause severe cardiotoxicity. While several mechanisms have been proposed to explain Dox‐induced cardiomyopathy (DIC), strategies to prevent it remain limited. In previous research on isolated cardiomyocytes, we identified that Empagliflozin (EMPA), an antidiabetic drug, mitigated Dox‐induced ER stress and apoptosis. In this in vivo study using rats, we further investigated EMPA's potential in preventing and treating DIC. Rats administered a cumulative dose of 15 mg/kg Dox exhibited significant cardiovascular damage, including left ventricular cavity dilation, decreased left ventricular ejection fraction (LVEF), ER dilation, mitochondrial defects and vacuole formation. These structural changes were linked to the activation of ER‐stress pathways (PERK, IRE1 and ATF6) and upregulation of apoptotic proteins initiated by ER stress. When EMPA (10 mg/kg/day) was administered either prophylactically or concurrently with Dox, it significantly attenuated adverse LV remodelling and preserved LVEF. Additionally, EMPA prevented ER stress and subsequent apoptosis in the myocardium of the Dox + EMPA‐treated group. These findings suggest that EMPA offers cardioprotective benefits in DIC, likely through the inhibition of ER‐stress‐induced myocardial injury.

## 1 Introduction

Doxorubicin (Dox) is an anthracycline chemotherapy drug widely used in the treatment of a variety of cancers including breast cancer, soft tissue sarcoma, leukaemia and lymphoma [1, 2]. Although Dox has been proven to increase the survival rate of cancer patients, its use is complicated by cardiotoxic side effects that can significantly affect the quality of life of cancer survivors, especially children. The cardiotoxic spectrum of Dox ranges from an asymptomatic decrease in left ventricular ejection fraction (LVEF) to end stage heart failure [3].

Dox‐induced cardiomyopathy (DIC) has been associated with an increase in oxidative stress (OS), lipid peroxidation, mitochondrial dysfunction, calcium overload, disruption of endoplasmic reticulum (ER) structure and function, and/or activation of several programmed cell death pathways leading to cardiomyocyte loss [4, 5]. Disturbed ER homeostasis causes the accumulation of unfolded and misfolded proteins in the ER lumen known as ER‐stress. This activates the unfolded protein response (UPR) via ER transmembrane proteins including protein‐kinase like ER kinase (PERK), activating transcription factor 6α (ATF6α) and inositol requiring kinase 1α (IRE1α) [6]. PERK, IRE1α and ATF6α dissociate from glucose‐regulated protein 78 (GRP78) and become activated, inducing transcription factors to increase chaperones and foldases to assist in protein folding. The UPR also helps in the attenuation of further protein synthesis and to reestablish homeostasis. Transcription factors, including X Box binding protein—1, play a critical role in the upregulation of chaperones like glucose regulated protein 94 (GRP94), GRP78, protein disulfide‐isomerase (PDI) and other antioxidant enzymes [7, 8]. It has been reported that under prolonged or severe stress due to Dox, UPR factors may fail to restore homeostasis and initiate ER‐stress induced programmed cell death such as apoptosis with the help of mitochondria [9, 10]. However, the intermediary role of ER‐stress in DIC is of increasing interest.

Various cardioprotective strategies including renin‐angiotensin system (RAS) antagonists, beta blockers and statins have been tested mainly for the prevention of DIC with the ultimate goal of improving the quality of life of cancer survivors [11, 12]. In recent years, an antidiabetic drug, Empagliflozin (EMPA), has received much attention in the heart failure setting [13, 14, 15]. EMPA is a sodium‐glucose co‐transporter 2 (SGLT‐2) inhibitor that helps maintain blood glucose levels in individuals with diabetes mellitus (DM) [16]. Several recent randomised controlled clinical trials with EMPA have reported a decrease in the risk of hospitalisation for heart failure and cardiovascular death in both diabetic and nondiabetic patients [17, 18, 19, 20]. Basic science studies have also reported the pleiotropic effects of EMPA, including the mitigation of OS, inflammation and apoptosis [21, 22, 23, 24]. In the present study, we examined the role of EMPA on cardiac structure and function with a focus on Dox‐induced ER stress.

## 2 Methods and Methods

### 2.1 Experimental Animal Model

A total of 30 adult male Sprague Dawley rats (200 ± 10 g) were randomly assigned to receive the following drug regimens: (i) Control [saline, six equal doses of 2.5 mg/kg body weight (BW) for 3 weeks]; (ii) EMPA (10 mg/kg/daily BW) via oral gavage; (iii) Dox [six equal doses of 2.5 mg/kg BW for 3 weeks with a total cumulative dose of 15 mg/kg BW intraperitoneally (IP)] and (iv) Dox + EMPA group, received prophylactic treatment with EMPA 1 week prior to the treatment of Dox. EMPA was administered at a dose of 10 mg/kg/day, based on previous preclinical studies demonstrating effective glycemic control and organ‐protective effects in murine models of diabetes and cardiovascular disease. This dose is commonly used in rodent studies to achieve plasma concentrations that approximate therapeutic levels observed in humans, accounting for species‐specific pharmacokinetic differences. EMPA treatment continued until the end of the experiment; that is, 6 weeks in both the EMPA and Dox + EMPA groups. All rats were maintained on a 12 h light–dark cycle and had access to standard chow and water ad libitum. All animal procedures were performed in accordance with ARRIVE guidelines as well as outlined by the Canadian Council on Animal Care. The University of Manitoba's Animal protocol review committee approved the study design and all study procedures (Protocol #22–012 AC11739). After 6 weeks of treatment, the rats were anaesthetised using ketamine (90 mg/kg) and xylazine (10 mg/kg) IP, euthanised by cardiac excision and the hearts were then rinsed in 0.9% saline. Heart samples were used for histological and protein analyses.

Groups:

1. *Control Group (n = 6)*
  - Received six saline injections
2. *EMPA Group (n = 6)*
  - Received 10 mg/kg BW EMPA via daily gavage
3. *Dox Group (n = 9)*
  - Received 2.5 mg/kg BW Dox via IP injections (six times)
4. *Dox + EMPA Group (n = 9)*
  - Received 10 mg/kg BW EMPA via daily gavage
  - Received 2.5 mg/kg BW Dox via IP injections (six times)

### 2.2 Hemodynamics

Rats underwent 1 day of blood pressure (BP) training with a noninvasive tail cuff method (CODA system, Kent Scientific, Torrington CT) to acclimatise them to the tail cuff system prior to experimental readings. This method was then used to measure BP at baseline, weeks 1, 4 and 6 in nonsedated and restrained rats. A total of 20 consecutive readings were taken at 1‐min intervals, and mean scores with a minimum of nine true readings were used for data analysis. Systolic BP (SBP), diastolic BP (DBP) and mean arterial pressure (MAP) parameters were measured. For blood glucose levels, the AlphaTRAK blood glucose monitoring system (Zoetis, USA) was used. Random blood glucose values were obtained once every week in nonsedated, restrained rats.

### 2.3 Echocardiography

To assess cardiovascular remodelling and cardiac function, transthoracic echocardiography was performed at baseline, weeks 1, 4 and 6. Rats were anaesthetised using isoflurane inhalant anaesthesia before taking images. Images were captured using a linear array ultrasound probe (13 MHz—Vivid 7, version 11.2, GE Medical Systems, Milwaukee, WI, USA) and analysed using the GE EchoPAC PC software. Parasternal short axis images were analysed using M‐mode to calculate heart rate (HR), interventricular septal wall thickness (IVS), posterior wall thickness (PWT), LV internal end‐diastolic diameter (LVIDd) and LVEF, as previously described [25]. The echocardiographic observers were blinded to the various treatment groups.

### 2.4 Histological Analysis

Histological analyses were performed according to lab‐established procedures on processed LV tissue. For light microscopy, tissue was sectioned and fixed in 10% neutral buffered saline for no more than 5 days before embedding in paraffin for imaging. Serial sections from each heart were cut 5 μm thick, then dewaxed, rehydrated and stained with either haematoxylin and eosin (H&E) or Masson's trichrome solution. H&E staining was performed to assess cardiac structural changes. Masson's trichrome stains normal myofiber red and collagen blue, allowing for the detection of fibrosis. Images were examined at 40× using the Cytation5 (BioTek Agilent, USA). Identical exposure settings were used for all digital images taken.

For electron microscopy (EM), tissue was sectioned and cut into 0.5 mm squares, fixed in 1:1 0.2 M phosphate buffer followed by a 2‐h postfixation in 1% osmium tetroxide in 0.1 M phosphate buffer at room temperature. Dehydration of tissues was performed in increasing ethanol concentrations then embedded in Epon 812.214. Tissue sections were stained with uranyl acetate and lead citrate. Tissue section grids were coded without prior knowledge of the source to eliminate bias. A Phillips CM12 electron microscope was used to determine the degree of cellular integrity. Histology scores were 0–5, with 0 being no tissue injury and 5 representing severe damage. Mann–Whitney and Kruskal–Wallis tests were used for nonparametric analysis of scores between each group [25].

### 2.5 Protein Estimation and Western Blots

To prevent protein degradation during the isolation of total cellular protein, LV tissue samples were flash frozen in liquid nitrogen then crushed and homogenised in a radioimmunoprecipitation assay lysis buffer with phosphatase inhibitor (Product # A32957, Thermo Scientific) and protease inhibitor cocktail (Roche Diagnostics, Canada). Samples were sonicated using a water sonicator for 20 min then centrifuged to remove cell debris, and supernatants were collected. Total protein estimation was performed on the supernatant using BSA as a standard according to the modified Bio‐Rad procedure. Microwell plate was then read using reader Cytation5, BioTek, Agilent, USA.

Sodium dodecyl sulfate polyacrylamide gel electrophoresis (SDS‐PAGE) was used for protein separation in each sample. SDS‐PAGE was performed on the samples in a 5% stacking gel and 8%, 10%, 12% or 15% resolving gel for 90 min at 70 mA at room temperature. The gel was then transferred to a 0.2 μm pore size polyvinylidene difluoride (PVDF) membrane (Product #: 88520, Thermo Scientific) for 1 h 45 min at constant 200 mA at 4°C. Posttransfer, membranes were blocked with 5% skim milk powder or BSA in 1× Tris Buffered Saline with 0.1% Tween 20 (Product # 0777‐1L, VWR) for 60 min at room temperature then probed using specific primary antibody overnight at 4°C. The samples were probed with Horseradish peroxidase conjugated goat‐antirabbit/antisecondary antibody (Product #: 1706515, BioRad) for 60 min at room temperature before band visualisation using ChemDoc imager (BioRad, Canada). Signals were detected using ECL Plus kit (Perkin Elmer, Canada). Primary antibodies used included: Anti‐Caspase‐12 (Abcam, #ab62484); Anti‐KDEL (Abcam, #ab176333), which recognises GRP94, GRP78 and PDI; Anti‐XBP1(Abcam, #ab37152); Anti‐ATF6 (Proteintech, #24169‐1‐AP); Anti‐PERK (Proteintech, #20582‐1‐AP); Anti‐IRE1α (Proteintech, #27582‐1‐AP); Anti XBP1 spliced and unspliced (Abcam, #ab37152); Anti‐SGLT2 (ab37296); Anticleaved caspase‐3 (Cell signalling, #9661); Anti‐JNK1 + JNK2 + JNK3 (Abcam, #ab179461); Anti‐JNK1 + JNK2 + JNK3 phospho (T183 + T183 + T221) (Abcam, #ab124956).

### 2.6 Statistical Analysis

Data are expressed as means ± SEM. Treatment groups were compared by one‐way analysis of variance (ANOVA), and Tukey–Kramer's test was performed to identify differences between the groups using GraphPad PRISM 8.0 software (RRID:SCR_002798). *p* ≤ 0.05 was considered to be significant.

## 3 Results

### 3.1 Survival and Changes in Body Weight, Blood Glucose Levels and Mean Blood Pressure

**FIGURE 1:**
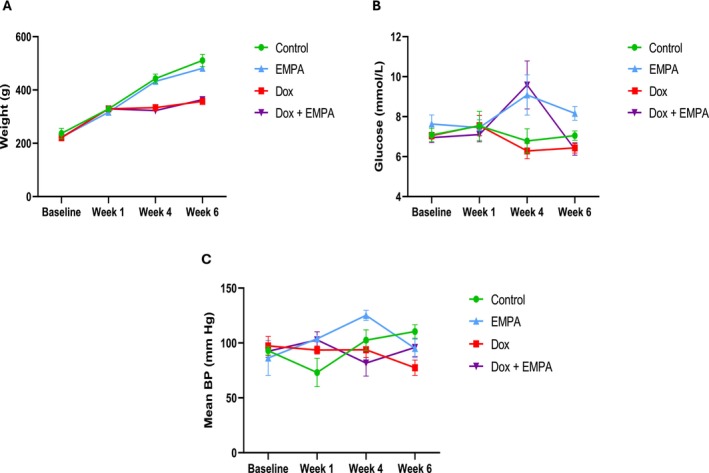
Effects of different treatments on body weight, glucose levels and mean blood pressure. (A) Body weight, (B) Blood glucose levels and (C) Mean blood pressure measurements throughout the experiment period in different treatment groups at baseline, Week 1, 4 and 6. Each experimental group consisted of six animals (*n* = 6).

Rats in all study groups had comparable BW at baseline and all experimental groups had a significant increase in weight from baseline. Rats in the control and EMPA groups were healthy and gained weight exponentially (Figure 1A) without any fluid retention. Dox alone treated rats had fluid retention in their abdominal cavity at the end of the experiment. Dox and Dox + EMPA had significant weight loss post‐Dox treatment, and EMPA had no effect on weight gain. At the end of 5 weeks, there was one death each in the Dox and Dox + EMPA group. There was another early death at the end of 3 weeks in the Dox + EMPA group; however, this was unrelated to the treatment. These deaths were not temporally associated with drug administration and were not preceded by clinical signs consistent with DIC. Additionally, the events were isolated and did not occur in a consistent pattern, suggesting that the mortality was incidental rather than treatment related. Both control and EMPA group had 100% survival until the experimental endpoint.

**FIGURE 2:**
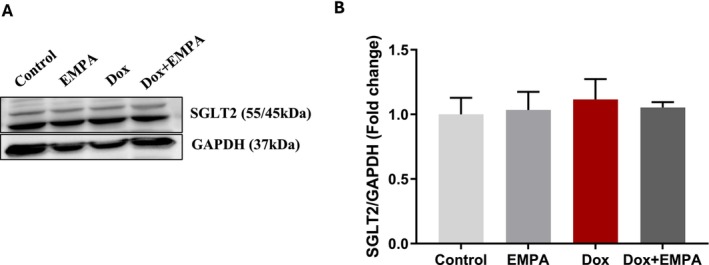
Expression of SGLT2 in whole heart. (A) WB of SGLT2 protein using SGLT2 antibody (1:500). GAPDH is shown as a loading control; (B) Densitometric analysis of SGLT2/GAPDH. Data is represented as means ± SD from three individual biological samples (*n* = 3).

At baseline, all groups had comparable fasting blood glucose levels of 7–8 mmol/L. There was an increase in blood glucose levels at Week 4 in the EMPA and Dox + EMPA groups as compared to the control and Dox groups (not significant) (Figure 1B). Interestingly, at Week 6, both the Dox and Dox + EMPA groups had significantly lower blood glucose levels compared to the EMPA group (*p* < 0.05); however, there were no significant changes compared to the control group (Table 1) (Figure 1B). As EMPA works through inhibiting SGLT2, we also recorded the protein expression of SGLT2 in the whole heart of all four groups (Figure 2A). There was no significant difference in the protein levels of SGLT2 between the treatment groups (Figure 2B).

**TABLE 1:** List of hemodynamic and echocardiographic parameters at baseline and Week 6 in different treatment groups.

Parameters	Timepoint	Control	EMPA	DOX	DOX + EMPA
Bodyweight (grams)	Baseline	237 ± 47	223 ± 5	218 ± 8	224 ± 5
	Week 6	510 ± 57^****^	481 ± 22^****^	387 ± 26*** ^**aabbbb**^	359 ± 37**** ^**aabbbb**^
Glucose (mmol/L)	Baseline	7.08 ± 0.81	7.63 ± 1.11	6.97 ± 0.54	8.81 ± 0.58
	Week 6	7.05 ± 0.57	8.16 ± 0.85	6.35 ± 0.65^**b**^	6.34 ± 0.67^**b**^
Systolic BP (mm Hg)	Baseline	114 ± 12	125 ± 18	118 ± 14	114 ± 22
	Week 6	137 ± 15	120 ± 24	100 ± 11^*a^	119 ± 25
Diastolic BP (mm Hg)	Baseline	83 ± 10	94 ± 17	88 ± 10	83 ± 19
	Week 6	98 ± 16	87 ± 14	67 ± 21^*a^	85 ± 20
Mean BP (mm Hg)	Baseline	93 ± 10	86 ± 18	97 ± 39	92 ± 20
	Week 6	111 ± 15	95 ± 17	77 ± 16^*a^	96 ± 21
HR (beats per minute)	Baseline	402 ± 8	405 ± 8	410 ± 11	411 ± 9
	Week 6	405 ± 6	402 ± 5	426 ± 42	407 ± 10
IVS (mm)	Baseline	1.25 ± 0.06	1.18 ± 0.02	1.17 ± 0.06	1.22 ± 0.04
	Week 6	1.26 ± 0.05	1.21 ± 0.33	1.20 ± 0.07	1.23 ± 0.02
PWT (mm)	Baseline	1.27 ± 0.04	1.26 ± 0.04	1.21 ± 0.04	1.25 ± 0.06
	Week 6	1.25 ± 0.03	1.26 ± 0.04	1.22 ± 0.04	1.26 ± 0.06
LVIDd (mm)	Baseline	7.48 ± 0.06	7.33 ± 0.08	7.33 ± 0.14	7.34 ± 0.08
	Week 6	7.51 ± 0.09	7.41 ± 0.02	10.41 ± 0.09**** ^**aaaabbbb**^	8.72 ± 0.17*** ^**aaaabbbbcccc**^
LVEF %	Baseline	79 ± 3	80 ± 1	81 ± 2	80 ± 2
	Week 6	79 ± 3	80 ± 2	50 ± 2*** ^**aaaabbbb**^	66 ± 3*** ^**aaaabbbbcccc**^

*Note:* The values are presented as mean ± SD. Each experimental group consisted of six animals (*n* = 6). *p < 0.05 versus baseline; ***p < 0.0005 versus baseline; ****p < 0.00005 versus baseline; ^a^ *p* < 0.05 versus control; ^aa^ *p* < 0.05 versus control; ^aaaa^ *p* < 0.0005 versus control; ^b^ *p* < 0.0005 versus control; ^bbbb^ *p* < 0.0005 versus EMPA; ^cccc^ *p* < 0.0005 versus Dox.

Abbreviations: BP, blood pressure; HR, heart rate; IVS, interventricular septal wall thickness; LVEF, left ventricular ejection fraction; LVIDd, left ventricular internal end‐diastolic diameter; PWT, posterior wall thickness.

At the end of the 6‐week experiment, both SBP and DBP were significantly lower in the Dox group (*p* < 0.05) compared to its baseline and the control group (Table 1). At Week 4, mean BP was significantly lower in the Dox and Dox + EMPA group (*p* < 0.05) as compared to the EMPA group, primarily due to elevated DBP in the EMPA group. However, at the end of the experiment, the change in mean BP was only significantly lower in the Dox group (*p* < 0.05) as compared to the control group (Figure 1C).

### 3.2 EMPA Improves Dox‐Induced Cardiovascular Remodelling

**FIGURE 3:**
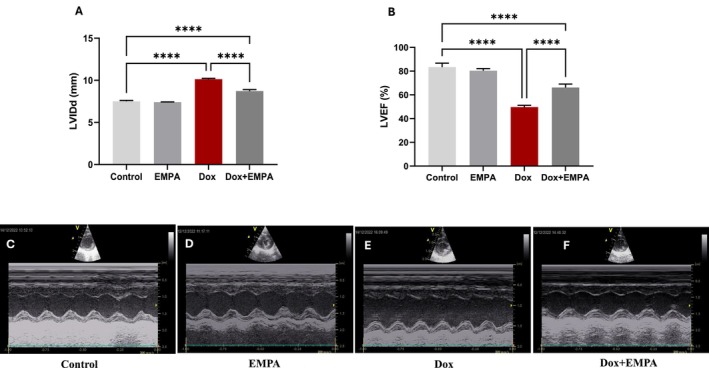
Effects of EMPA on Dox induced cardiovascular remodelling. (A) LVIDd (mm) and (B) LVEF (%) as determined by M‐mode echocardiography. Each experimental group consisted of six animals (*n* = 6). (C–F) representative M‐mode images of LV cavity dimensions of different groups. Data is represented as mean ± SD. ****p < 0.00005.

Cardiovascular remodelling in all four groups was assessed noninvasively by echocardiography (Table 1). At baseline and 6 week follow‐up, there were no significant changes observed in HR, IVS and PWT. At the end of Week 6, left ventricular end diastolic diameter (LVIDd) was significantly increased in Dox treated animals (*p* < 0.0005) as compared to both control and EMPA groups (Figure 3A,E). Prophylactic and continuous administration of EMPA in the Dox + EMPA group showed significant improvement in the LVIDd compared to the Dox group at 6 weeks (*p* < 0.0005) (Figure 3A,F). Additionally, in the Dox treated group, there was a significant decline in LVEF (*p* < 0.0005) compared to both the control and EMPA groups (Figure 3B). However, LV systolic function was preserved in the Dox + EMPA group with a significant increase as compared to the Dox group (*p* < 0.0005).

### 3.3 Cardiac Ultrastructural Changes and Fibrosis

**FIGURE 4:**
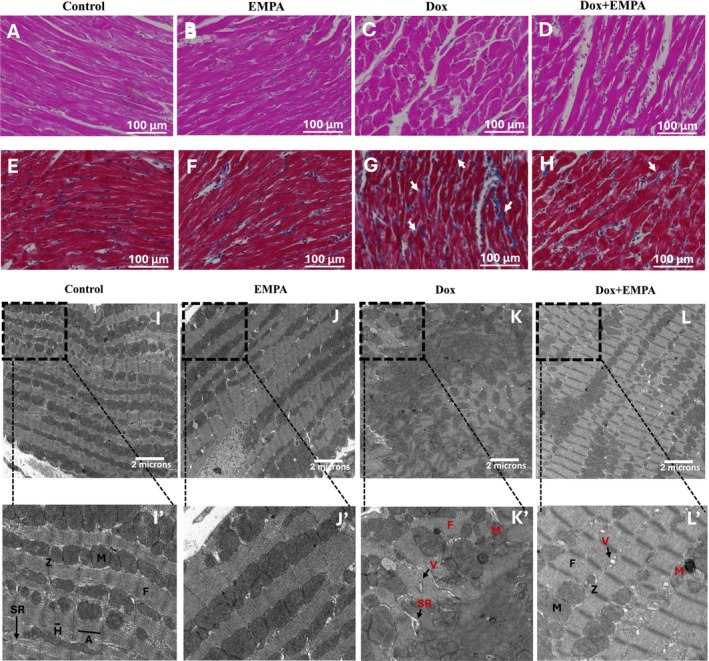
Effects of treatment on ultrastructure of heart and collagen deposition. (A–D) Representative images of cardiac sections stained with haematoxylin and eosin staining. Images were obtained at 40×, scale bar = 100 μm. (E–H) Representative images of cardiac sections stained with Masson's trichome staining. Images were obtained at 40×, scale bar = 100 μm. (I–L) Representative electron microscopy images of heart sample. Direct magnification = 5800X; Scale bar = 2 μm. (I′–L′) Zoomed images of respective image on the top panel. A, A‐band; F, myofibrils; H, H‐band; M, mitochondria; SR, sarcoplasmic reticulum; V, vacuole; Z, Z‐line.

H&E staining was used to stain cardiac tissue and assess morphology. Both the control and EMPA group demonstrated normal rod‐shaped like morphology with normal striations and elongated nucleus (Figure 4A,B). In contrast, the Dox group showed a significant disruption of the myocardial structure from rod‐shaped to circular, alteration in the shape of nucleus, disarray as well as loss of myofibrils (Figure 4C). The cardioprotective effects of EMPA were observed via reduced disruption of the myocardial structure with intact nuclei and striated myocardium present (Figure 4D). Masson's trichome staining confirmed significant fibrosis in the Dox group (Figure 4G) compared to both control and EMPA groups (Figure 4E,F), as indicated by white arrows. In the Dox + EMPA group, however, there was no significant cardiac fibrosis with findings comparable to the control group (Figure 4H). EM revealed normal myocardial structure in both the control and EMPA groups. In both groups, there were normal mitochondria with abundant regular cristae and myofibril architecture that were well organised. Myofilaments had striations comprised of A‐bands, H‐bands with intersecting Z‐lines (Figure 4I′,J′). However, the Dox treated group showed increased fragmentation and lysis of the myofibrils, loss of ER reticular network and mitochondrial deformity. We also observed dilated sarcoplasmic reticulum (SR) as well as vacuolisation (Figure 4K′) in the Dox group. Myofibrils in the Dox group did not have distinguished Z‐lines either. The Dox + EMPA group also demonstrated vacuolisation and dilated SR; however, the mitochondria and myofibrils were more intact compared to the Dox alone group. Z‐lines were also intact as well as A‐bands and H‐bands in Dox + EMPA group compared to the Dox group (Figure 4L′).

### 3.4 Dox Mediated Changes on ER‐Stress Signalling Proteins and Effects of EMPA

**FIGURE 5:**
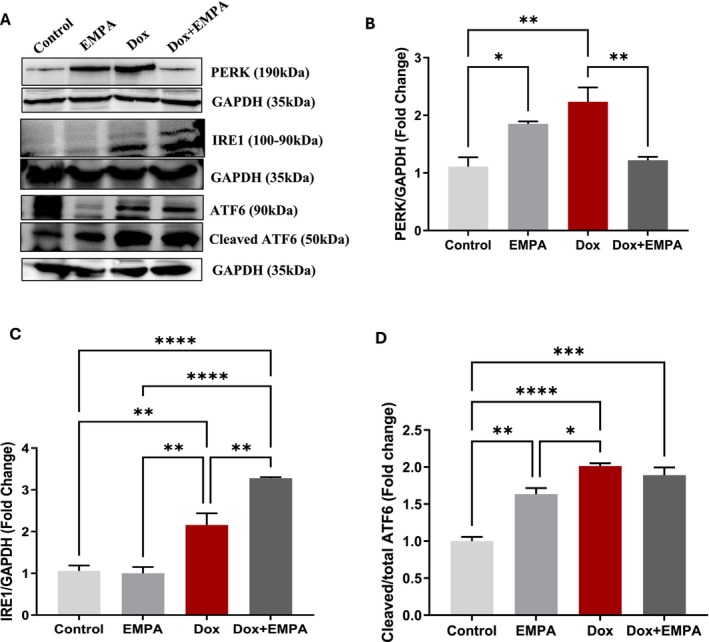
Effects of different treatments on UPR signalling proteins. (A) WB of respective proteins using protein PERK antibody (1:500), IRE1α antibody (1:2000), ATF6α antibody (1:5000); GAPDH was used as a loading control; (B–D) Densitometric analysis of PERK/GAPDH, IRE1/GAPDH, Cleaved/total ATF6, respectively. Data represented are means ± SD from three individual biological samples (*n* = 3). *p < 0.05; **p < 0.005; ***p < 0.0005; ****p < 0.00005.

As ER‐stress signalling proteins (PERK, IRE1, ATF6α) are responsible for inducing UPR and to bring back ER homeostasis, we analysed their protein expression using WB. Both Dox and EMPA groups had a significant increase in PERK as compared to the control group (*p* < 0.005, *p* < 0.05, respectively) (Figure 5A,B). Interestingly, the Dox + EMPA group had a significantly low (*p* < 0.005) expression of PERK in whole heart (Figure 5B) as compared to both EMPA and Dox group. In contrast, IRE1 expression had low to no expression in both control and EMPA groups. Dox caused a significant increase in IRE1 (*p* < 0.005) as compared to both control and EMPA groups (Figure 5A,C). However, Dox + EMPA group showed a significant increase in IRE1 compared to all other groups (*p* ≤ 0.005) (Figure 5A,C). On the other hand, ATF6 activation and its cleaved fragment (ATF6 50 kDa) were significantly higher in the Dox group (*p* < 0.00005) as compared to the control and EMPA groups (*p* < 0.05). However, both EMPA and Dox + EMPA groups, also significantly activated the ATF6 cleavage and overexpressed cleaved ATF6 as compared to the control group (Figure 5A,D).

### 3.5 Effects of EMPA on ER Chaperones

**FIGURE 6:**
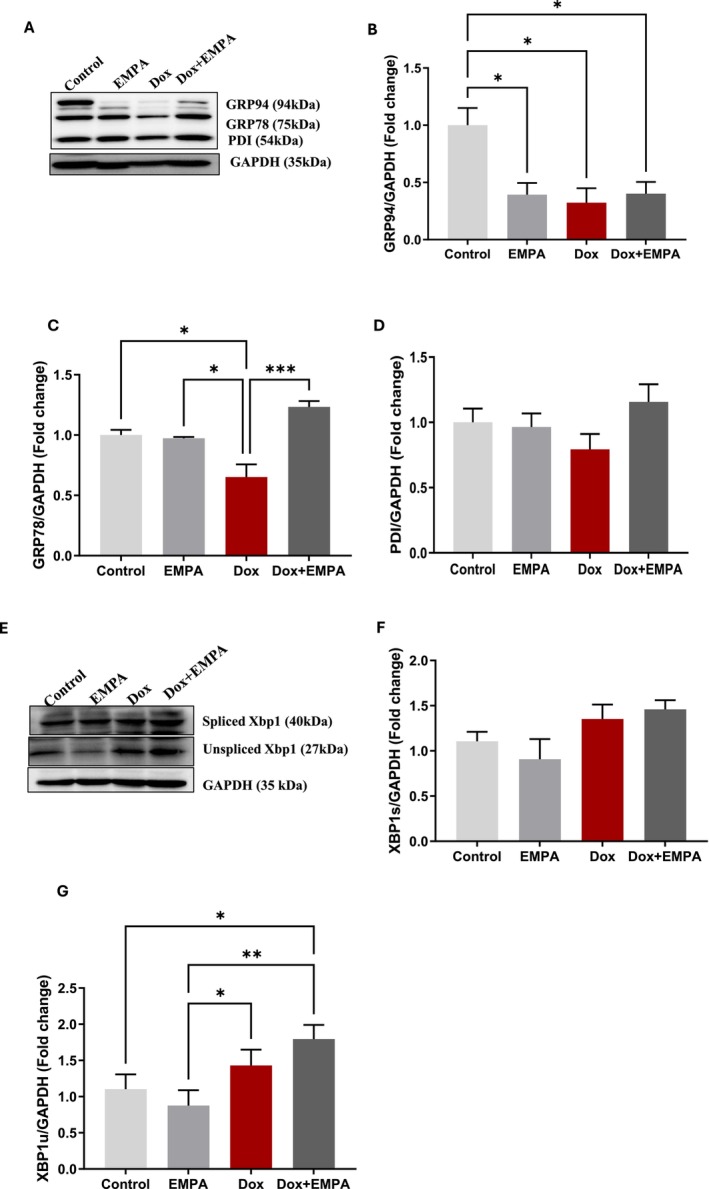
Protein expression of UPR chaperones and transcription factor X‐box binding protein 1 (XBp1). (A) WB of respective proteins using KDEL antibody (1:1000); GAPDH is shown as a loading control. (B–D) Densitometric analysis of GRP94/GAPDH, GRP78/GAPDH, PDI/GAPDH, respectively. (E) WB of respective proteins using XBP1 antibody (1:500); GAPDH is shown as a loading control; (F and G) Densitometric analysis of spliced XBP1/GAPDH, unspliced XBP1/GAPDH, respectively. Data is represented as means ± SD from three individual biological samples (*n* = 3). *p < 0.05; **p < 0.005; ***p < 0.0005.

As different UPR proteins were expressed (Figure 6), we also measured protein expression of ER chaperones that are activated and expressed upon UPR induction. Although GRP94 was highly expressed in the control group, all other groups had significantly lower expression (*p* < 0.05) (Figure 6A,B). The chief UPR chaperone, GRP78, was significantly downregulated in Dox treatment (*p* < 0.05) compared to the EMPA and control groups. However, in the Dox + EMPA group, GRP78 was significantly upregulated (*p* < 0.0005) compared to the Dox alone group (Figure 6A,C). No significant difference was observed in the expression of PDI in either group (Figure 6D). The protein expression of transcription factor spliced XBP1 and its negative regulator unspliced XBP1 were also recorded. Although we observed protein expression of spliced XBP1 in all treatment groups, there was no significant difference between the groups (Figure 6E,F). On the other hand, unspliced XBP1 was significantly upregulated in both Dox and Dox + EMPA groups (Figure 6E,G) compared to the EMPA group (*p* ≤ 0.05). However, when compared to the control group, unspliced XBP1 was upregulated in the Dox + EMPA group only (*p* < 0.05).

### 3.6 ER‐Stress Initiated Apoptosis and Its Mitigation by EMPA

**FIGURE 7:**
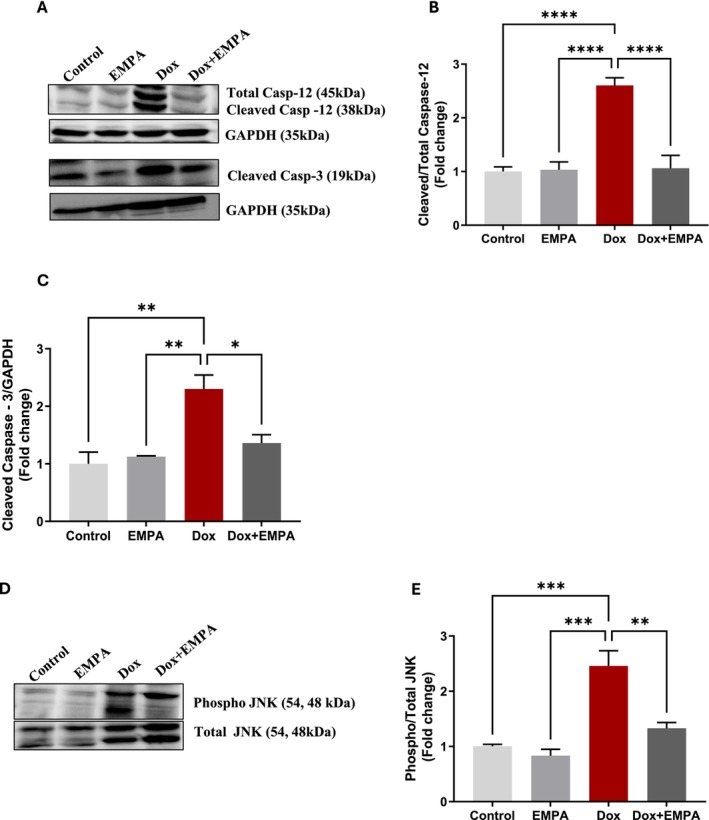
ER‐stress induced apoptosis and its mitigation by EMPA. (A and D) WB of respective proteins using caspase‐12 antibody (1:1000); cleaved caspase‐3 antibody (1:1000); phospho‐JNK antibody (1:500); total JNK antibody (1:2000); GAPDH was used as a loading control. (B, C and E) Densitometric analysis of cleaved caspase −12/caspase −12, Caspase‐3/GAPDH, Phospho/total JNK, respectively. Data is represented as means ± SD from three individual biological samples (*n* = 3). *p < 0.05; **p < 0.005; ***p < 0.0005; ****p < 0.00005.

When UPR inducers fail to restore ER functioning, ER‐stress induced apoptosis is initiated via phosphorylation of JNK and cleaved caspases. There was a 2.5‐fold increase in ER‐resident cleaved caspase‐12/total caspase‐12 in the Dox group compared to control and EMPA (*p* < 0.0005). Interestingly, EMPA treatment in the Dox + EMPA group was able to significantly lower the activation of caspase‐12 (Figure 7A,B) compared to the Dox group (*p* < 0.0005). Additionally, in the Dox group, there was a 2.5‐fold increase in cleaved caspase‐3 expression compared to the control and EMPA groups (*p* < 0.005). However, cleaved caspase‐3 was significantly decreased in the Dox + EMPA group compared to the Dox group (*p* < 0.05) (Figure 7A,C). Finally, we recorded the expression of phospho/total JNK. Phospho/total JNK was highly upregulated in the Dox group (2.5‐fold increase) compared to control and EMPA groups (*p* < 0.0005) (Figure 7D,E). EMPA treatment in the Dox + EMPA group was able to significantly downregulate the expression of phospho/total JNK compared to the Dox group (*p* < 0.005). This downregulation of phospho/total JNK in the Dox + EMPA group was not significantly different from the control group (Figure 7E).

## 4 Discussion

Despite several attempts to prevent and treat DIC, the rate of its development in the cancer population has not improved over the years. Although traditional therapies including RAS antagonists and beta blockers are used in the management of heart failure [11], no drug has been approved specifically for the treatment of DIC and heart failure. Even though EMPA has been approved as guideline directed medical therapy for heart failure [13, 14, 15, 17, 18, 19, 20], little is known of its potential role in the setting of DIC.

In this study using a chronic in vivo model of DIC, we found that prophylactic and concurrent treatment with EMPA can prevent adverse cardiovascular remodelling. Specifically, there were adverse changes in the structure and function of the myocardium in rats treated with Dox, which were rescued by EMPA treatment. Additionally, we recorded activation of all three ER transmembrane proteins, but not chaperones, indicating a switch from the adaptive response to apoptosis following Dox treatment. EMPA was able to maintain ER homeostasis in our in vivo DIC model through initiation of the ER adaptive response, upregulated chaperone expression and downregulated ER‐initiated apoptosis proteins.

### 4.1 Cardiovascular Remodelling

SGLT2i are the latest addition to guideline‐directed medical therapy in the management of heart failure as they have the potential to improve cardiovascular remodelling [13, 14, 15, 17, 18, 19, 20]. Previous murine studies have evaluated the role of EMPA, a SGLT2i, in the prevention of DIC [21, 26, 27]. Bariş et al. administered 18 mg/kg BW Dox to Sprague Dawley rats on Days 2, 4, 8,10, 12 and 14 alongside 10 mg/kg BW EMPA daily during this 2‐week period. They observed that Dox‐only treated rats had an increase in LV volumes with a depressed LVEF of 35% at the 2‐week study end point. Conversely, rats concurrently treated with EMPA + Dox had a significant reduction in LV volumes with an improved LVEF of 74% [26]. Additional studies have confirmed dapagliflozin (DAPA), another SGLT2i, in the improvement of LVEF in rats treated with DAPA + Dox [28, 29]. In their study, Chang et al. reported that prophylactic treatment with DAPA 10 mg/kg BW daily for 6 weeks followed by 4 weeks of Dox at 20 mg/kg improved LVEF to 85% as compared to 75% in the Dox only group [28]. Similarly, Hseih et al. also showed a significant improvement in LVEF to 75% when DAPA was administered 0.1 mg/kg daily for 4 weeks concurrently with 12 mg/kg BW weekly injections of Dox compared to 50% LVEF in the Dox only group [29]. All of the aforementioned studies used a SGLT2i in either the prophylactic setting or concurrent treatment with Dox. In our preclinical study, we mimicked a clinically relevant chronic in vivo DIC model in SD rats with EMPA being administered before, during and after Dox therapy. Our 6‐week study demonstrated that Dox + EMPA significantly improved LV remodelling with a decrease in LV cavity dimensions by approximately 20% as compared to the Dox‐only treated group. There was also a significant improvement in LVEF of 66% in the Dox + EMPA treated rats as compared to a LVEF of 50% in the Dox only group which corroborates the findings of the previous aforementioned studies [21, 26, 27]. It is worth noting that significant BW reduction can influence cardiac function. However, severe or rapid weight loss accompanied by ascites is often associated with worsened cardiac outcomes in Dox‐treated groups [30]. In our study, although the Dox + EMPA group exhibited significant weight loss (Table 1), this was accompanied by improved cardiac markers and absence of abdominal edema, suggesting that EMPA's cardioprotective effects extend beyond the impact of weight change alone. Overall, SGLT2i used in both the prevention and treatment settings can prevent adverse cardiovascular remodelling in preclinical models of DIC.

### 4.2 Histological Analysis

In addition to LV cavity dilation and LV systolic dysfunction, loss of myocardial integrity is an established side effect of Dox [31, 32, 33]. Several preclinical studies using a chronic DIC model have reported myofibril fragmentation, mitochondrial swelling, loss of ER reticular network and vacuolisation of the cytoplasm [33, 34, 35]. Recently, EMPA was shown to be beneficial in preserving myocardial ultrastructure in several preclinical studies in either a diabetic cardiomyopathy or postmyocardial infarction model [36, 37]. In the DIC model, Bariş et al. observed that a SGLT2i can maintain mitochondrial integrity with preserved myofibrils in Dox + EMPA treated rats using EM [27]. Our study also demonstrated the cardioprotective effects of EMPA in preserving cardiomyocyte ultrastructure using EM. In our comprehensive study, we also performed light microscopy which confirmed that EMPA was able to reduce cardiac fibrosis in the Dox + EMPA group. Two previous studies have reported similar findings that EMPA mitigates Dox induced fibrosis in a C57BL/6 mouse model [21, 23]. The effects of EMPA on reducing cardiac fibrosis has been previously reported in other disease models as well including hypertension [14], diabetes [38] and postmyocardial infarction [39]. Overall, SGL2Ti, including EMPA, reduce cardiac fibrosis and preserve mitochondrial integrity and myofibrils in a DIC model.

### 4.3 UPR and ER‐Initiated Apoptosis

The intermediary role of ER‐stress leading to cardiac dysfunction and apoptosis has been previously proposed as an important mechanism of DIC by our group and others [10, 35, 40, 41]. Since EMPA has been reported to reduce OS and inflammation which can trigger UPR and ER‐initiated apoptosis, we investigated the potential effects of EMPA in reducing ER‐stress and subsequently DIC.

We observed the activation of all three ER transmembrane proteins following Dox treatment indicating the initiation of ER‐stress. A significant upregulation of ATF6 and IRE1α under Dox treatment was previously reported by our group and others, in both in vitro and in vivo models of DIC [42]. Although EMPA treatment in the Dox + EMPA group did not have any significant effect on ATF6 activity, IRE1α and its downstream molecule unspliced XBP1 were significantly upregulated. Additionally, a significant increase in IRE1α expression in the Dox + EMPA group played a pivotal role in restoring ER homeostasis and promoting cell survival under Dox‐induced stress. This is supported by the restored morphological and functional aspects of the heart observed in our experiments (Figures 3 and 4). While previous reports have identified general ER stress reduction by SGLT2 inhibitors [43, 44], our data uniquely reveal that EMPA promotes adaptive UPR signalling via enhanced XBP1 unspliced protein, thereby distinguishing its mechanism from broader ER stress modulators. Additionally, unspliced XBP1 forms a complex with spliced XBP1 to suppress its function and promote ER‐stress recovery [45, 46].

In EMPA group, our findings suggest that EMPA initially activates PERK signalling to induce protective chaperones, followed by a resolution phase characterised by downregulation of PERK and sustained IRE1α/XBP1 activity in Dox + EMPA group. This temporal modulation aligns with models proposed by Hetz and Papa, where dynamic UPR signalling determines cell fate under chronic ER stress [6, 8]. EMPA‐induced inhibition of PERK and subsequent autophagy has been reported in an ischemia/reperfusion myocardial injury model as well [35]. On the other hand, other studies reported an increase in autophagy following EMPA treatment that helped in attenuating ER stress and subsequent apoptosis [15, 44]. Similar contradictory results were found in our study as EMPA treatment alone increased PERK expression and possibly cardioprotective autophagy. Although we observed overexpression of all three UPR proteins in the Dox group, the expression of the ER chaperone GRP78 was significantly low in the Dox treated group. GRP78 in the whole heart is essential in downregulating overall ER stress and inhibiting ER‐initiated apoptosis [7, 35, 47]. In our study, EMPA upregulated the expression of the ER chaperone, GRP78, in the Dox + EMPA group which may play an essential role in cardioprotection via assisting protein folding and inhibiting overall ER stress.

We also measured ER‐linked apoptotic protein expression in different treatment groups. ER‐resident caspase‐12 is activated by cleavage and leads to cleavage of caspase‐3 and links ER‐initiated apoptosis to mitochondrial apoptosis [48, 49]. Both caspase‐12 and 3 were highly upregulated in the Dox group and EMPA treatment was able to rescue apoptosis in the Dox + EMPA group. In addition to activation of caspases, we also observed phosphorylation of JNK which has been reported as one of the key players in Dox‐induced apoptosis that can be activated by both ER‐dependent and ER‐independent pathways [40, 50]. The activation of these cell executioners in the Dox group led to myocardial loss as confirmed by histology. The role of EMPA in attenuating apoptosis has been reported in multiple studies in several disease models [14, 15, 49]. However, the effects of EMPA on procaspase‐12 downregulation to block ER‐initiated apoptosis are very important in attenuating cardiomyocyte death and promoting healthy myocardium.

In conclusion, our results indicate that EMPA induced IRE1α/XBP1 signalling helped manage the overall ER‐stress and aided in ER‐stress recovery in the Dox + EMPA group. Our findings also suggest that early administration may offer cardioprotective benefits to DIC. However, further clinical studies are needed to determine the safety and efficacy of EMPA before such an approach can be integrated into standard clinical care.

## 5 Future Directions

Our study demonstrates the involvement of IRE1 in maintaining ER homeostasis and attenuating ER‐initiated apoptosis in in vivo settings. There are a few chemical IRE1 inhibitors available such as STF‐083010, ORIN1001 and toxoflavin that can be utilised to better understand and to compare the role of IRE1 in Dox‐induced ER stress‐initiated apoptosis and to compare the role of EMPA in inducing its cardioprotective effects via IRE1 adaptive response. Additionally, siRNA‐mediated knockdown of IRE1α or XBP1 could be used to evaluate their critical roles in EMPA‐mediated cardio protection and to further elucidate the underlying molecular pathways.

Future studies should also incorporate more comprehensive morphological assessments, including gross and histological evaluation of cardiac dilation, to further support the functional and hemodynamic findings. In addition, histological staining of heart sections for markers of apoptosis and necrosis would provide spatial evidence of cellular injury and further strengthen the mechanistic understanding of the cardioprotective effects observed.

## Author Contributions

**David C. Y. Cheung:** investigation, writing – original draft, methodology, writing – review and editing, software, project administration, formal analysis, data curation. **D. Allison Ledingham:** investigation, writing – original draft, methodology, writing – review and editing, software, formal analysis, project administration, data curation. **Pawan K. Singal:** conceptualization, investigation, funding acquisition, methodology, validation, visualization, writing – review and editing, writing – original draft, software, formal analysis, project administration, data curation, supervision, resources. **Davinder S. Jassal:** conceptualization, investigation, funding acquisition, writing – original draft, methodology, validation, visualization, writing – review and editing, software, formal analysis, project administration, resources, supervision, data curation. **Akshi Malik:** conceptualization, investigation, funding acquisition, writing – original draft, methodology, validation, visualization, writing – review and editing, software, formal analysis, project administration, data curation, supervision, resources. **Danielle Desautels:** writing – original draft, writing – review and editing, formal analysis, project administration, supervision. **Joerg Herrmann:** writing – original draft, writing – review and editing, formal analysis, project administration, supervision.

## Funding

This work was supported by the Heart and Stroke Foundation of Canada, St. Boniface Hospital Foundation and Molson Women’s Heart Health Program.

## Conflicts of Interest

The authors declare no conflicts of interest.

## Data Availability

The data that support the findings of this study are available from the corresponding author upon reasonable request.

## References

[1] E. A. Lefrak , J. Piťha , S. Rosenheim , and J. A. Gottlieb , “A Clinicopathologic Analysis of Adriamycin Cardiotoxicity,” Cancer 32 (1973): 302–314. 4353012 10.1002/1097-0142(197308)32:2<302::aid-cncr2820320205>3.0.co;2-2

[2] K. Johnson‐Arbor and R. Dubey , “Doxorubicin,” in StatPearls (StatPearls Publishing, 2021).

[3] P. K. Singal and N. Iliskovic , “Doxorubicin‐Induced Cardiomyopathy,” New England Journal of Medicine 339 (1998): 900–905. 9744975 10.1056/NEJM199809243391307

[4] P. Singal , T. Li , D. Kumar , I. Danelisen , and N. Iliskovic , “Adriamycin‐Induced Heart Failure: Mechanisms and Modulation,” Molecular and Cellular Biochemistry 207 (2000): 77–86. 10888230 10.1023/a:1007094214460

[5] D. Cappetta , A. de Angelis , L. Sapio , et al., “Oxidative Stress and Cellular Response to Doxorubicin: A Common Factor in the Complex Milieu of Anthracycline Cardiotoxicity,” Oxidative Medicine and Cellular Longevity 2017 (2017): e1521020. 10.1155/2017/1521020 PMC5664340 29181122

[6] C. Hetz and F. R. Papa , “The Unfolded Protein Response and Cell Fate Control,” Molecular Cell 69 (2018): 169–181. 29107536 10.1016/j.molcel.2017.06.017

[7] A. S. Lee , “The ER Chaperone and Signaling Regulator GRP78/BiP as a Monitor of Endoplasmic Reticulum Stress,” Methods 35 (2005): 373–381. 15804610 10.1016/j.ymeth.2004.10.010

[8] C. Hetz , “The Unfolded Protein Response: Controlling Cell Fate Decisions Under ER Stress and Beyond,” Nature Reviews. Molecular Cell Biology 13 (2012): 89–102. 22251901 10.1038/nrm3270

[9] L. Zhao and S. L. Ackerman , “Endoplasmic Reticulum Stress in Health and Disease,” Current Opinion in Cell Biology 18 (2006): 444–452. 16781856 10.1016/j.ceb.2006.06.005

[10] F. Yarmohammadi , R. Rezaee , A. W. Haye , and G. Karimi , “Endoplasmic Reticulum Stress in Doxorubicin‐Induced Cardiotoxicity May Be Therapeutically Targeted by Natural and Chemical Compounds: A Review,” Pharmacological Research 164 (2021): 105383. 33348022 10.1016/j.phrs.2020.105383

[11] A. Ludke , A. A.‐R. Al‐Shudiefat , S. Dhingra , D. Jassal , and P. Singal , “A Concise Description of Cardioprotective Strategies in Doxorubicin‐Induced Cardiotoxicity,” Canadian Journal of Physiology and Pharmacology 87 (2009): 756–763. 19898559 10.1139/Y09-059

[12] S. Dellapasqua , P. T. Aliaga , E. Munzone , et al., “Pegylated Liposomal Doxorubicin (Caelyx) as Adjuvant Treatment in Early‐Stage Luminal B‐Like Breast Cancer: A Feasibility Phase II Trial,” Current oncology 28 (2021): 5167–5178. 34940072 10.3390/curroncol28060433 PMC8700739

[13] I. Andreadou , P. Efentakis , E. Balafas , et al., “Empagliflozin Limits Myocardial Infarction in Vivo and Cell Death in Vitro: Role of STAT3, Mitochondria, and Redox Aspects,” Frontiers in Physiology 8 (2017): 1077. 29311992 10.3389/fphys.2017.01077 PMC5742117

[14] H.‐C. Lee , Y. L. Shiou , S. J. Jhuo , et al., “The Sodium‐Glucose Co‐Transporter 2 Inhibitor Empagliflozin Attenuates Cardiac Fibrosis and Improves Ventricular Hemodynamics in Hypertensive Heart Failure Rats,” Cardiovascular Diabetology 18 (2019): 45. 30935417 10.1186/s12933-019-0849-6 PMC6444638

[15] N. Nasiri‐Ansari , C. Nikolopoulou , K. Papoutsi , et al., “Empagliflozin Attenuates Non‐Alcoholic Fatty Liver Disease (NAFLD) in High Fat Diet Fed ApoE(−/−) Mice by Activating Autophagy and Reducing ER Stress and Apoptosis,” International Journal of Molecular Sciences 22 (2021): 818. 33467546 10.3390/ijms22020818 PMC7829901

[16] G. Chawla and K. K. Chaudhary , “A Complete Review of Empagliflozin: Most Specific and Potent SGLT2 Inhibitor Used for the Treatment of Type 2 Diabetes Mellitus,” Diabetes and Metabolic Syndrome: Clinical Research & Reviews 13 (2019): 2001–2008. 10.1016/j.dsx.2019.04.035 31235127

[17] B. Zinman , C. Wanner , J. M. Lachin , et al., “Empagliflozin, Cardiovascular Outcomes, and Mortality in Type 2 Diabetes,” New England Journal of Medicine 373 (2015): 2117–2128. 26378978 10.1056/NEJMoa1504720

[18] M. Packer , S. D. Anker , J. Butler , et al., “Cardiovascular and Renal Outcomes With Empagliflozin in Heart Failure,” New England Journal of Medicine 383 (2020): 1413–1424. 32865377 10.1056/NEJMoa2022190

[19] M. Böhm , J. Butler , G. Filippatos , et al., “Empagliflozin Improves Outcomes in Patients With Heart Failure and Preserved Ejection Fraction Irrespective of Age,” Journal of the American College of Cardiology 80 (2022): 1–18. 35772911 10.1016/j.jacc.2022.04.040

[20] D. von Lewinski , E. Kolesnik , N. J. Tripolt , et al., “Empagliflozin in Acute Myocardial Infarction: The EMMY Trial,” European Heart Journal 43 (2022): 4421–4432. 36036746 10.1093/eurheartj/ehac494 PMC9622301

[21] J. Sabatino , S. de Rosa , L. Tammè , et al., “Empagliflozin Prevents Doxorubicin‐Induced Myocardial Dysfunction,” Cardiovascular Diabetology 19 (2020): 66. 32414364 10.1186/s12933-020-01040-5 PMC7229599

[22] M. Pirklbauer , “Anti‐Inflammatory Potential of Empagliflozin,” Inflammopharmacology 29 (2021): 573–576. 33728540 10.1007/s10787-021-00797-9 PMC7997819

[23] V. Quagliariello , M. de Laurentiis , D. Rea , et al., “The SGLT‐2 Inhibitor Empagliflozin Improves Myocardial Strain, Reduces Cardiac Fibrosis and Pro‐Inflammatory Cytokines in Non‐Diabetic Mice Treated With Doxorubicin,” Cardiovascular Diabetology 20 (2021): 150. 34301253 10.1186/s12933-021-01346-y PMC8305868

[24] C.‐C. Wang , Y. Li , X. Q. Qian , et al., “Empagliflozin Alleviates Myocardial I/R Injury and Cardiomyocyte Apoptosis via Inhibiting ER Stress‐Induced Autophagy and the PERK/ATF4/Beclin1 Pathway,” Journal of Drug Targeting 30 (2022): 858–872. 35400245 10.1080/1061186X.2022.2064479

[25] C. R. Eekhoudt , T. Bortoluzzi , S. S. Varghese , et al., “Comparing Flaxseed and Perindopril in the Prevention of Doxorubicin and Trastuzumab‐Induced Cardiotoxicity in C57Bl/6 Mice,” Current Oncology 29 (2022): 2941–2953. 35621631 10.3390/curroncol29050241 PMC9139942

[26] C.‐Y. Wang , C. C. Chen , M. H. Lin , et al., “TLR9 Binding to Beclin 1 and Mitochondrial SIRT3 by a Sodium‐Glucose co‐Transporter 2 Inhibitor Protects the Heart From Doxorubicin Toxicity,” Biology 9 (2020): 369. 33138323 10.3390/biology9110369 PMC7693736

[27] V. Ö. Barış , A. B. Dinçsoy , E. Gedikli , S. Zırh , S. Müftüoğlu , and A. Erdem , “Empagliflozin Significantly Prevents the Doxorubicin‐Induced Acute Cardiotoxicity via Non‐Antioxidant Pathways,” Cardiovascular Toxicology 21 (2021): 747–758. 34089496 10.1007/s12012-021-09665-y

[28] W.‐T. Chang , J. Y. Shih , Y. W. Lin , et al., “Dapagliflozin Protects Against Doxorubicin‐Induced Cardiotoxicity by Restoring STAT3,” Archives of Toxicology 96 (2022): 2021–2032. 35438302 10.1007/s00204-022-03298-y

[29] P.‐L. Hsieh , P. M. Chu , H. C. Cheng , et al., “Dapagliflozin Mitigates Doxorubicin‐Caused Myocardium Damage by Regulating AKT‐Mediated Oxidative Stress, Cardiac Remodeling, and Inflammation,” International Journal of Molecular Sciences 23 (2022): 10146. 36077544 10.3390/ijms231710146 PMC9456438

[30] A. M. Zobeydi , S. Mousavi Namavar , M. Sadeghi Shahdani , et al., “Mitigating Doxorubicin‐Induced Hepatotoxicity in Male Rats: The Role of Aerobic Interval Training and Curcumin Supplementation in Reducing Oxidative Stress, Endoplasmic Reticulum Stress and Apoptosis,” Scientific Reports 15 (2025): 6604. 39994295 10.1038/s41598-025-91133-6 PMC11850886

[31] G. Akolkar , A. K. Bagchi , P. Ayyappan , D. S. Jassal , and P. K. Singal , “Doxorubicin‐Induced Nitrosative Stress Is Mitigated by Vitamin C via the Modulation of Nitric Oxide Synthases,” American Journal of Physiology. Cell Physiology 312 (2017): C418–C427. 28100487 10.1152/ajpcell.00356.2016

[32] P. K. Singal , N. Siveski‐Iliskovic , M. Hill , T. P. Thomas , and T. Li , “Combination Therapy With Probucol Prevents Adriamycin‐Induced Cardiomyopathy,” Journal of Molecular and Cellular Cardiology 27 (1995): 1055–1063. 7563102 10.1016/0022-2828(95)90074-8

[33] M. Kamel , L. Farouk , A. Osman , O. Khorshid , and M. Abdo , “Comparative Study of the Protective Effect of Metformin and Sitagliptin Against Doxorubicin‐Induced Cardiotoxicity in Rats,” Clinical Pharmacology & Biopharmaceutics 6 (2017).

[34] E. D. B. Danz , J. Skramsted , N. Henry , J. A. Bennett , and R. S. Keller , “Resveratrol Prevents Doxorubicin Cardiotoxicity Through Mitochondrial Stabilization and the Sirt1 Pathway,” Free Radical Biology & Medicine 46 (2009): 1589–1597. 19303434 10.1016/j.freeradbiomed.2009.03.011

[35] H. Y. Fu , S. Sanada , T. Matsuzaki , et al., “Chemical Endoplasmic Reticulum Chaperone Alleviates Doxorubicin‐Induced Cardiac Dysfunction,” Circulation Research 118 (2016): 798–809. 26838784 10.1161/CIRCRESAHA.115.307604

[36] J. Wang , X. Huang , H. Liu , et al., “Empagliflozin Ameliorates Diabetic Cardiomyopathy via Attenuating Oxidative Stress and Improving Mitochondrial Function,” Oxidative Medicine and Cellular Longevity 2022 (2022): 1122494. 35585884 10.1155/2022/1122494 PMC9110219

[37] M. Mizuno , A. Kuno , T. Yano , et al., “Empagliflozin Normalizes the Size and Number of Mitochondria and Prevents Reduction in Mitochondrial Size After Myocardial Infarction in Diabetic Hearts,” Physiological Reports 6 (2018): e13741. 29932506 10.14814/phy2.13741 PMC6014462

[38] B. Lin , N. Koibuchi , Y. Hasegawa , et al., “Glycemic Control With Empagliflozin, a Novel Selective SGLT2 Inhibitor, Ameliorates Cardiovascular Injury and Cognitive Dysfunction in Obese and Type 2 Diabetic Mice,” Cardiovascular Diabetology 13 (2014): 148. 25344694 10.1186/s12933-014-0148-1 PMC4219031

[39] G. Li , C. Zhao , and S. Fang , “SGLT2 Promotes Cardiac Fibrosis Following Myocardial Infarction and Is Regulated by miR‐141,” Experimental and Therapeutic Medicine 22 (2021): 1–9. 10.3892/etm.2021.10147 PMC8120516 34007324

[40] A. Malik , A. K. Bagchi , D. S. Jassal , and P. K. Singal , “Interleukin‐10 Mitigates Doxorubicin‐Induced Endoplasmic Reticulum Stress as Well as Cardiomyopathy,” Biomedicine 10 (2022): 890. 10.3390/biomedicines10040890 PMC9027958 35453640

[41] A. K. Bagchi , A. Malik , G. Akolkar , et al., “Study of ER Stress and Apoptotic Proteins in the Heart and Tumor Exposed to Doxorubicin,” Biochimica et Biophysica Acta 1868 (2021): 119039. 33857568 10.1016/j.bbamcr.2021.119039

[42] M. Lu , S. Merali , R. Gordon , et al., “Prevention of Doxorubicin Cardiopathic Changes by a Benzyl Styryl Sulfone in Mice,” Genes & Cancer 2 (2011): 985–992. 22701764 10.1177/1947601911436199 PMC3374628

[43] B. Tirosh , N. N. Iwakoshi , L. H. Glimcher , and H. L. Ploegh , “Rapid Turnover of Unspliced Xbp‐1 as a Factor That Modulates the Unfolded Protein Response *,” Journal of Biological Chemistry 281 (2006): 5852–5860. 16332684 10.1074/jbc.M509061200

[44] B. Radlinger , F. Hornsteiner , S. Folie , et al., “Cardioprotective Effects of Short‐Term Empagliflozin Treatment in Db/Db Mice,” Scientific Reports 10 (2020): 19686. 33184414 10.1038/s41598-020-76698-8 PMC7665199

[45] H. Yoshida , M. Oku , M. Suzuki , and K. Mori , “pXBP1(U) Encoded in XBP1 Pre‐mRNA Negatively Regulates Unfolded Protein Response Activator pXBP1(S) in Mammalian ER Stress Response,” Journal of Cell Biology 172 (2006): 565–575. 16461360 10.1083/jcb.200508145 PMC2063676

[46] M. Ala , M. R. F. Khoshdel , and A. R. Dehpour , “Empagliflozin Enhances Autophagy, Mitochondrial Biogenesis, and Antioxidant Defense and Ameliorates Renal Ischemia/Reperfusion in Nondiabetic Rats,” Oxidative Medicine and Cellular Longevity 2022 (2022): 1197061. 35126806 10.1155/2022/1197061 PMC8816566

[47] H. Tscheschner , J. Reinkober , P. Most , et al., “Glucose‐Regulated Protein 78 (GRP78) Overexpression Inhibits Doxorubicin Cardiotoxicity,” European Heart Journal 34 (2013): P5691.

[48] J. Hitomi , T. Katayama , M. Taniguchi , A. Honda , K. Imaizumi , and M. Tohyama , “Apoptosis Induced by Endoplasmic Reticulum Stress Depends on Activation of Caspase‐3 via Caspase‐12,” Neuroscience Letters 357 (2004): 127–130. 15036591 10.1016/j.neulet.2003.12.080

[49] H. Shiraishi , H. Okamoto , A. Yoshimura , and H. Yoshida , “ER Stress‐Induced Apoptosis and Caspase‐12 Activation Occurs Downstream of Mitochondrial Apoptosis Involving Apaf‐1,” Journal of Cell Science 119 (2006): 3958–3966. 16954146 10.1242/jcs.03160

[50] P. Haberzettl and B. G. Hill , “Oxidized Lipids Activate Autophagy in a JNK‐Dependent Manner by Stimulating the Endoplasmic Reticulum Stress Response,” Redox Biology 1 (2013): 56–64. 24024137 10.1016/j.redox.2012.10.003 PMC3757667

